# The microbiome’s hidden influence: preclinical insights into inflammatory responses in necrotizing enterocolitis

**DOI:** 10.1007/s00281-025-01059-4

**Published:** 2025-11-24

**Authors:** Briana M. Peterson, Ina Rudloff, Nadia S. Deen, Sara K. Di Simone, Ramesh M. Nataraja, Gergely Toldi, Maurizio Pacilli, Steven P. Garrick, Steven X. Cho, Marcel F. Nold, Samuel C. Forster, Claudia A. Nold-Petry

**Affiliations:** 1 Department of Paediatrics, Monash University, Melbourne, VIC Australia; 2 Ritchie Centre, Hudson Institute of Medical Research, Melbourne, VIC Australia; 3 Centre for Innate Immunity and Infectious Diseases, Hudson Institute of Medical Research, Melbourne, VIC Australia; 4 Department of Mathematics and Natural Sciences, BRAC University, Dhaka, Bangladesh; 5 Department of Paediatric Surgery, Monash Children’s Hospital, Melbourne, VIC Australia; 6 Liggins Institute, University of Auckland, Auckland, New Zealand; 7 Monash Newborn, Monash Children’s Hospital, Melbourne, VIC Australia; 8 Department of Molecular and Translational Sciences, Monash University, Melbourne, VIC Australia

**Keywords:** Necrotizing enterocolitis, Neonatal, Microbiome, Preclinical modeling

## Abstract

Necrotizing enterocolitis (NEC) is the most common surgical emergency in preterm infants; nonetheless, besides supportive measures, no treatment is available. NEC significantly increases length of hospitalization of preterm infants, causes severe morbidity and up to 70% mortality. Despite limited understanding of the underlying mechanisms, prematurity, dysbiosis and an underdeveloped immune system are known to increase the risks of developing NEC. The low weight of preterm infants (often < 2000 g) and unpredictable progression of NEC hinder clinical research; hence, most of our mechanistic understanding of NEC pathophysiology has arisen from animal models. Recent advances in bacterial genomic analyses highlighted the intestinal microbiome’s key role in NEC, strengthening the concept that this disease results from an interaction between the patient’s developing immune system and their microbiome. This notion is supported by the moderate effect of probiotics in preventing NEC. Here, we review the current knowledge on how the immune system interacts with the intestinal microbiome in early life, including in relation to NEC, describe the current evidence from cohort studies, clinical trials, in vivo and in vitro models used to study NEC, and methods to modulate the immune system and microbiome in early life. Knowledge on the early-life microbiome and immune system in health and diseases, including NEC, can be harnessed to develop novel and urgently needed immunomodulatory and microbiota-based therapeutics.

## Introduction

Globally, necrotizing enterocolitis (NEC) affects 3–7% of very low birth weight (≤ 1500 g) infants younger than 32–33 weeks gestational age (GA) [1–3], increasing to 12% in infants weighing 501–750 g at birth [4]. First described in 1823 [5, 6], NEC was thought to originate from nosocomial infections until the mid-20th century. The term “necrotizing enterocolitis” was introduced in 1965 to describe ‘an often fatal syndrome in preterm infants consisting of vomiting, abdominal distention, gastrointestinal bleeding and shock’ [7]. Bell’s 1978 three stage NEC classification [8], which was updated in 1986 [9], still remains in clinical use today [10].

Due to its non-specific early presentation and unpredictable progression, NEC is difficult to diagnose and manage [11]. By the time of NEC diagnosis, infants often present with sepsis or multi-organ failure [12]. Treatment is limited to bowel rest, broad-spectrum antibiotics and supportive care (e.g. blood pressure management, intravenous nutrition). Mortality ranges from 20 to 30%, rising to 70% for surgical NEC [1, 13, 14]. Survivors face significant risks of neurodevelopmental delays and short gut syndrome [14].

Despite intensive research, NEC pathogenesis remains poorly understood, hindering prevention and development of novel treatments. Usually, infants are sterile in utero and acquire their microbiome from birth [15] in parallel with their immune system development. Although incompletely mapped, disrupted interactions between the immune system and the microbiome development are implicated in NEC. This review outlines the current knowledge of NEC-related changes in the immune system and microbiome development, and evaluates contributions made by pre-clinical in vivo and in vitro models in advancing knowledge of NEC.

## Risk factors and general pathogenesis

Although the exact pathogenesis underlying the overwhelming inflammation and subsequent tissue injury that leads to NEC remains unclear, several risk factors have been identified: prematurity [16, 17], hypoxia [18], genetic predisposition [19], formula feeding [20], immature mucosal barrier integrity [21] and intestinal microbial imbalance (termed dysbiosis) [14, 22, 23].

Prematurity strongly associates with NEC incidence and severity [16, 17]: immature intestinal barrier function and motility potentially increase translocation of bacteria or their components, leading to excessive inflammation while the premature immune system is ill-equipped to combat pathogens in the extrauterine environment [23]. Single nucleotide polymorphisms in inflammatory cytokine receptors, including *VEGFC*−2578 A, *IL-18*−607 A and *IL-4RA*−1903 G, may increase inflammatory signaling and predispose infants to NEC [24]. Intermittent hypoxia from apnoea of prematurity (inversely correlated with GA) disrupts blood flow to the gut and increases the risk of tissue damage [18]. Formula feeding triples NEC risk [25], potentially due to the absence of protective compounds found in breastmilk (e.g. unbound free fatty acids [26], anti-inflammatory cytokines, immunoglobulins (Ig), growth factors, cellular components and commensal bacteria) [27, 28]. Moreover, the imbalance of the gut microbiome in NEC impairs immune homeostasis [29, 30] and will be one of the foci of this review.

## NEC management

Attempts to improve NEC management, through changes in rates and timing of enteral feeding [31], administration of colostrum [32, 33], erythropoietin [34, 35], corticosteroids [36], arginine [37], Ig [38, 39] or lactoferrin [40] have shown little if any improvement in clinical outcomes. Probiotics have shown promise in reducing NEC, but have yet to reach their full potential, as discussed below.

## NEC pathoimmunology

The pathoimmunology of NEC comprises innate and adaptive immune dysregulation [23]. As per the traditional paradigm, innate immunity dominates in early life, as the adaptive immune system slowly adjusts to the extrauterine environment [41].

### Innate responses

In preterm infants, innate immune responses often lack counter-regulation, leading to uncontrolled intestinal cytokine production, tissue damage and reduced repair [35]. Tissue damage impairs the intestinal barrier and allows bacterial translocation, further increasing recruitment of innate inflammatory cells and augmenting inflammatory responses [42].

#### Pattern recognition receptors (PRRs)

Innate immunity is activated via pattern recognition receptors (PRRs), which detect pathogen associated molecular patterns (PAMPs) and trigger pro-inflammatory signaling [43]. Toll-like receptors (TLRs), a PRR subtype expressed by immune and intestinal epithelial cells, are key to distinguishing beneficial commensal bacteria from pathogens and are implicated in NEC pathogenesis [43]. Dysregulation, especially increased TLR4 or TLR2 and insufficient counter-regulatory TLR9 signaling, promoted inflammation in murine NEC [22], while inhibition of TLR4 or its signaling reduced NEC [44–46]. TLR4 is activated by lipopolysaccharide (LPS), a cell wall component of gram-negative bacteria [47] including Pseudomonadota (formerly Proteobacteria) which are increased in and may promote NEC [48, 49], though definite evidence is lacking. LPS-induced TLR4 signaling activates NF-κB-mediated production of pro-inflammatory cytokines (e.g. IL-1, IL-6, IL-8, TNF (tumor necrosis factor), IFN-γ (interferon-γ)) and also reduces the number of protective Tregs (regulatory T-cells) [14, 22, 50]. Other TLR4-mediated responses, including necroptosis (inflammatory programmed cell death) [51] and autophagy (lysosomal degradation of intracellular components) [52], may contribute to NEC pathogenesis. Notably, in *Tlr4*-deficient mice, reduced expression of the necroptosis-associated kinases *Ripk1* and *Ripk3* inhibited necroptosis and NEC-associated inflammation [51]. Autophagy is prototypically not associated with excessive inflammation; however, TLR4 induced autophagy can contribute to NEC development via inhibited enterocyte migration within the murine ileum [52]. The expression of *TLR4* and *TLR2* is also greater in human fetal enterocytes than mature cells, predisposing premature infants to greater inflammatory responses [53].

Muramyl dipeptide, a ligand of the intracellular immune receptor NOD2, inhibited *Tlr4* expression in murine NEC, thereby reducing intestinal injury [54]. Expression of *Tlr9* and *Tlr4* were reciprocally related in murine embryonic development and TLR9 activation limited TLR4 signaling in enterocytes, preventing inflammation and NEC development [55]. NEC infant stool sequencing identified microbial community patterns supporting TLR4 activation and TLR9 under-stimulation [56]. Deficiencies in TLR4 signaling adaptors MyD88 and TRIF protected against murine NEC [35], while absence of *Tlr2*,* Tlr5* or *Tlr9* increased NEC susceptibility, suggesting complex PRR interactions [57]. Moreover, deficiency in IL-1R8 (aka SIGIRR), a negative TLR signaling regulator which is also reduced in fetal enterocytes vs. adult cells, is also associated with NEC [22].

In summary, PRRs contribute to NEC pathogenesis, highlighting the role of environmental factors (e.g. microbial composition) in excessive inflammation characteristic in NEC.

#### Pro- and anti-inflammatory mediators

Gene expression of innate and adaptive immune response mediators is developmentally regulated [58], including via epigenetic mechanisms [59], predisposing preterm infants to excessive inflammation and NEC [22, 53, 59]. For example, in comparison to mature enterocytes, fetal cells have increased expression of pro-inflammatory genes such as *TRAF6*, *NFKB1* and *MYD88* [53], and increased TNF abundance in NEC tissues contributes to intestinal injury through apoptosis and reactive oxygen species [60].

Immature enterocytes also have lower abundance of IκB, an important NF-κB inhibitor that reduces pro-inflammatory mediators including IL-8 [61]. Further, in a rat NEC model, NF-κB blockade improved survival and tissue injury [62]. Anti-inflammatory genes, including *TNFAIP3* and *TOLLIP*, were also reduced in fetal enterocytes compared to mature cells– and expression was even lower in enterocytes of NEC infants [53].

Anti-inflammatory cytokines like IL-10 and IL-37 reduce NEC severity in mice [63] and the intestinal IL-37 receptor is more abundant in non-NEC controls compared to NEC infants [22].

Overall, these findings suggest that developmental down-regulation of inflammation is disrupted in NEC, leading to excessive innate immune activation, partially initiated by intestinal TLR activation, production of pro-inflammatory mediators and impaired counter-regulatory anti-inflammatory mechanisms.

### Adaptive responses

The adaptive immune system responds to specific antigens processed and presented by antigen-presenting cells, like macrophages. Upon antigen recognition and co-stimulation, naïve CD4⁺ T cells differentiate based on the antigen and local cytokine milieu. In NEC, reduced TGF-β_2_ led to increased intestinal macrophage infiltration and activation [64], potentially contributing to elevated T cell responses. Pregnancy skews the maternal and fetal/neonatal adaptive immune system towards type 2 polarization, protecting the hemi-allograft fetus from rejection [41]. In NEC, however, polarization shifts towards type 3, with increased IL-17 producing Th17 and type 3 innate lymphoid cells (ILC3s) [50] while IL-22 levels were reduced [22, 65]. Other sources of IL-22, such as unconventional CD8^+^ γδ T-cells, were also reduced in NEC [66]. Pro-inflammatory *IL36A*, *IL36B*, and *IL36G* were increased in NEC [22] and IL-36γ-deficient mice were protected from T-cell driven intestinal inflammation [67]. IL-36γ may also inhibit Treg development [68], and anti-inflammatory FoxP3^+^ Tregs were 60% lower in NEC infants’ ileal lamina propria compared to age-matched healthy controls [69]. Altogether, NEC is characterized by a skew towards inflammatory type 3 responses and a deficiency of anti-inflammatory Tregs, driven by dysregulated antigen processing, presentation, and T cell activation.

## The intestinal microbiome in early life

During delivery and in the days and weeks after birth, the infant faces one of its biggest challenges: the first exposure to microbes. Despite reports of bacteria present in amniotic fluid [70], placenta [71], umbilical cord blood, fetal membranes [72] and meconium [70, 73], a comprehensive study of term and preterm placentas found that, except for *Streptococcus agalactia*, a pathogen that was detected in 5% of samples due to perinatal infection, bacteria were either acquired through contamination during labour/delivery or through laboratory reagents [74]. After birth, the intestinal microbiome diversifies and stabilizes during the first years of life, with commensal microbiota forming a symbiotic relationship with the host [75, 76]. Thus, to maintain health, a delicate balance between immune tolerance of commensal bacteria and effective control of pathogens must be achieved.

### The intestinal microbiome in healthy infants

The initial colonizers of the healthy neonatal intestine include mainly facultative anaerobes [77, 78]. Between 3 and 12 weeks of life, the microbiome begins a period of diversification [79], including colonization with obligate anaerobes such as *Clostridium*,* Bifidobacterium* and *Bacteroides* species [75]. Gradual diversification of the microbiome continues until stabilization at around three years of age, when it resembles the adult microbiome [80].

In the first month after birth, the mode of delivery and the external environment (e.g. feeding, medications, locations) are the main drivers of microbiome composition [81]. Infants delivered through cesarean sections (C/S) are typically colonized by *Enterococcus faecalis*,* Enterococcus faecium*,* Staphylococcus epidermidis*,* Streptococcus parasanguinis*,* Klebsiella oxytoca*,* Klebsiella pneumoniae*,* Enterobacter cloacae* or *Clostridium perfringens* [76, 81]. For vaginally delivered infants, the intestinal microbiome is enriched in *Bifidobacterium*,* Escherichia* and *Bacteroides* [76, 81]. Prior to weaning, either full or partial breastmilk-feeding has the most pronounced effect on microbiome composition, with babies receiving breastmilk having a higher abundance of *Bifidobacterium* compared to exclusively formula-fed babies [76]. Maternal prenatal antibiotic therapy also impacts microbiome composition, with reduced *Bacteroides vulgatus* and increased *Escherichia coli*,* Klebsiella oxytoca*,* Klebsiella pneumoniae* and *Enterobacter cloacae* [81]. This is consistent with postnatal antibiotic administration, including reduced species richness after meropenem, cefotaxime and ticarcillin-clavulanate, and a dominance of multidrug-resistant members of *Escherichia*,* Klebsiella* and *Enterobacter* genera in infants [82]. Early alterations in diversity or relative abundance of microbes were associated with a broad range of later adverse health outcomes including obesity, inflammatory bowel disease, atopic disease, type 2 diabetes and behavioural disorders [83–85].

### The intestinal microbiome in preterm infants

Since feeding establishment is often delayed in preterm infants [86], it should be considered as a common confounder when comparing the early-life microbiome to term infants. In comparison to term infants, preterm infants are reported to have an increased proportion of facultative anaerobes such as *Enterobacteriaceae*,* Enterococcaceae* and *Lactobacilli*, but fewer obligate anaerobes such as *Bifidobacteria*,* Bacteroides* and *Atopobium* [87]. At the class level, the bacterial composition in stool samples of very low birth weight preterm infants progressed from Bacilli to Gammaproteobacteria to Clostridia with age regardless of antibiotics, feeds or delivery mode, indicating that host biology also drives microbial colonization in the newborn [79]. Additionally, preterm infants showed increased abundance of potentially pathogenic hospital-associated bacteria including *Escherichia coli*,* Enterococcus faecalis* and *Klebsiella pneumoniae*, independent of birth weight, feeding mode or antibiotic treatment [88]. These findings support evidence that prolonged hospital stays, often unavoidable in most infants born < 35 weeks GA, substantially affect microbiome acquisition [81].

### The intestinal microbiome in infants with NEC

It was first proposed in 1979 that the hospital environment may hinder normal microbial colonization and lead to NEC [89]. In 2001, this hypothesis was refined in recognition of data showing that colonization patterns may contribute to NEC [90]. Despite extensive research over the last decade as detailed in Table 1, no single microbe has been identified as the cause of NEC.

Applying culturing, 16S rRNA profiling and metagenomic sequencing of stool samples, most but not all [88, 91–95] studies identified differences in the intestinal microbiome across taxonomic levels between preterm infants with and without NEC. Increased Pseudomonadota [96–100] and Gammaproteobacteria [97, 101–103] alongside decreased Bacillota [97, 103, 104] and Actinomycetota [98, 100, 103, 105] are commonly reported in NEC. Many species have been reported as more abundant in or associated with NEC, including *Clostridium spp. (C. perfringens*,* C. butyricum*,* C. neonatale*) [102, 106–110], *Klebsiella pneumoniae* [106, 111–114], *Cronobacter sakazakii *(previously *Enterobacter sakazakii*, formula contaminate) [107], *Citrobacter koseri* [111], *Corynebacterium striatum* [115], *Morganella morganii* [115], *Staphylococcus aureus* [98, 102] and *Enterococcus faecalis* [114], while other genera are less abundant in NEC infants, including *Lactobacillus* [91, 99, 100, 111, 116, 117], *Bifidobacterium* [98, 100, 105, 113], *Streptococcus* [91, 99, 105, 114, 118] and *Veillonella* [118, 119]. Contradictory findings are common regarding the NEC microbiome. For example, while specific species such as *K. pneumoniae*, *E. faecalis* and *Staphylococcus aureus* associate with NEC, their genera *Klebsiella*, *Enterococcus* and *Staphylococcus*, respectively, are often less abundant in NEC infants compared to controls [102, 116, 120, 121].

These discrepancies could result from small sample sizes [115, 122, 123] or varying GA, bodyweight at time of sampling, NEC severity, feeding practices and use of medication between studies [93, 99, 103, 105, 114]. Sample collection protocols and procedural differences of microbiome analysis, including sample collection timing, 16S ribosomal RNA amplicon region and culture techniques, may also contribute to observation variations (Table 1) [124]. In addition to intrinsic bacteria factors, the timing of acquisition likely plays a role in determining the immune system’s reaction to specific microbes. Longitudinal studies have identified that the microbiome is less stable [125] and reduces in diversity prior to NEC development [104, 105, 111, 113, 123]. Thus, it is likely no single species is the driver of NEC, rather impaired patterns of microbiome acquisition and resulting dysbiosis alter inflammatory responses.

**Table 1:** Published studies on the intestinal microbiome in infants with NEC from 2015 to 2025 where n number is greater than one. Stool was sampled in all studies, except Romano-Keeler (2018) and Stewart (2019) in which frozen or formalin-fixed paraffin-embedded intestinal tissues were used, respectively. Abbreviations: necrotizing enterocolitis (NEC), ribosomal ribonucleic acid (rRNA), operational taxonomic units (OTUs), quantitative polymerase chain reaction (qPCR), denaturing gradient gel electrophoresis (DGGE), Temporal temperature gradient gel electrophoresis (TTGE), small intestine perforation (SIP)

Reference	Clinical and procedural features					Observations (compared to controls unless stated otherwise)
	GA at birth (weeks)	Birth weight (g)	Sample time	*n* numbers	Technology used	
Sim (2015) [106]	< 32	< 1,250	Pre diagnosis	12 NEC 44 controls	16S rRNA gene sequencing and culture	Overabundance of a *Clostridium* OTU and a *Klebsiella* OTU in NEC infants. Presence of *Clostridium perfringens* type A confirmed by culture in all samples in which *Clostridium* OTU was found.
Zhou (2015) [107]	24 to 31	< 1,860	Pre and post diagnosis	12 NEC 26 controls	16S rRNA gene sequencing	At the genus level, *Clostridium sensu stricto* significantly higher in early onset NEC cases and *Escherichia*/*Shigella* and *Cronobacter* higher in late onset NEC cases.
McMurty (2015) [118]	≤ 34	≤ 1,500	Post or pre diagnosis	21 NEC 74 controls	16S rRNA gene sequencing	Two classes, Actinomycetota and Clostridia, significantly lower in NEC specimens. Abundances of two bacterial genera, *Veillonella* and *Streptococcus*, significantly lower NEC specimens across all three Bell’s stages.
Cassir (2015) [108]	28 to 32	1000 to 1750	Post diagnosis	15 NEC 15 controls	16S rRNA pyrosequencing; culture-based methods and qPCR	*Clostridium butyricum* associated with NEC using molecular and culture-based methods or qPCR. *Staphylococci* less prevalent in NEC samples.
Leach (2015) [115]	24 to 32	< 1,500	Pre diagnosis	4 NEC 18 controls	16S rDNA gene sequencing	No significant difference in fecal communities between NEC and non-NEC infants. Potentially pathogenic bacteria, such as *Corynebacterium striatum* and *Morganella morganii*, detected significantly more in infants with NEC.
Heida (2016) [109]	24 to 29	560 to 1735	Pre diagnosis	11 NEC 22 controls	16S rRNA gene sequencing	Higher abundance of *Clostridium perfringens* and *Bacteroides dorei* in meconium and stool samples a week prior to NEC diagnosis. Equivalent control samples were more dominated by *Staphylococci*.
Abdulkadir (2016) [95]	23 to 30	500 to 1470	Pre and post diagnosis	10 NEC 10 controls	qPCR	No difference in the bacterial load of stool samples collected from infants over a period of 2 weeks prior to and post NEC diagnosis. No difference in the bacterial load of stool samples from NEC infants and non-NEC infants.
Warner (2016) [101]	24.7 to 28.7	≤ 1,500	Pre diagnosis	28 NEC 94 controls	16S rRNA gene sequencing	At the class level, increased proportions of Gammaproteobacteria and lower proportions of both Negativicutes and combined Clostridia–Negativicutes in infants who developed NEC.
Ward (2016) [119]	23 to 29	415 to 1741	Pre diagnosis	27 NEC 117 controls	Shotgun metagenomic sequencing	Microbial diversity of NEC stool samples was similar to preterm controls prior to day 17 of life. After day 17 of life, NEC stool samples showed less *Veillonella* and increased *Escherichia coli*. Presence of *E.coli* in at least one stool sample correlated with NEC-associated death.
Stewart (2016) [125]	23 to 30	500 to 1810	Pre and post diagnosis	7 NEC 28 controls	16S rRNA gene sequencing	The microbial composition of stool from NEC infants is less stable than that of control infants.
Barron (2017) [93]	-	≤ 1,500	Pre diagnosis	30 NEC 65 controls	16S rRNA pyrosequencing	No significant differences evident between control and NEC, medically treated NEC, and surgically treated NEC and in pre-NEC and post-NEC samples.
Roze (2017) [102]	24 to 31	-	Post diagnosis	16 NEC 78 controls	16 S rRNA gene sequencing	*Clostridium sensu stricto* and Gammaproteobacteria associated with NEC.
					Culture	Association between *Clostridium* at the genus level and *Clostridium neonatale* and *Staphylococcus aureus* at the species level with NEC. Lower proportion of *Klebsiella* and *Citrobacter* observed in NEC infants.
Dobbler (2017) [111]	≤ 32	< 2,050	Pre diagnosis	11 NEC 29 controls	16S rRNA gene sequencing, shotgun metagenomic sequencing	*Citrobacter koseri* and/or *Klebsiella pneumoniae* are dominating taxa in NEC. Low diversity and low abundance of *Lactobacillus* correlate with NEC risk in preterm infants.
Wandro (2018) [122]	23 to 31	620 to 1570	Pre and post diagnosis	3 NEC 21 controls	qPCR	Overall microbial abundance was lower in NEC infant stool samples than controls.
					16S rRNA gene sequencing	No microbial composition signature was linked to NEC.
Itani (2018) [116]	27 to 35	< 2,250	Pre and post diagnosis	11 NEC 11 controls	qPCR, TTGE and culture	No significant difference in microbiota between NEC and non-NEC by culture method and TTGE. By qPCR, significant differences with a higher colonization by *Staphylococci* and lower colonization by *Enterococci* and *Lactobacilli* in NEC compared to healthy controls prior to NEC onset.
Romano-Keeler (2018) [110]	29 mean	1,274	Post diagnosis	12 NEC 14 controls	16S rRNA gene sequencing	Microbial diversity was lower in NEC tissue samples. Higher abundances of *Staphylococcus* and *Clostridium sensu stricto*, and lower abundances of *Actinomyces* and *Corynebacterius* in NEC.
Stewart (2019) [96]	25 median	731 median	Post diagnosis	13 NEC 16 controls (SIP)	16S rRNA gene sequencing	Microbial diversity was lower in NEC tissue samples than SIP tissue samples. Pseudomonadota relative abundance was higher in NEC tissue samples. Bacteroidota relative abundance was higher in SIP tissue samples.
Liu (2019) [123]	28 to 33	773 to 2149	Pre and post diagnosis	4 NEC 17 controls	16S rRNA gene sequencing	Microbial diversity reduced from birth to NEC diagnosis. Control stool samples exhibited more stable microbiome composition than NEC stool samples pre-diagnosis. *Enterococcus*,* Streptococcus* and *Peptoclostridium* genera were more abundant in NEC stool samples.
Feng (2019) [91]	> 28	< 2,700	Post diagnosis	16 NEC 16 controls	16S rRNA gene sequencing	No significant difference in the total diversity of microbiota between NEC cases and controls. *Propionibacterium* more abundant in NEC infants; *Lactobacillus*, *Phascolarctobacterium* and *Streptococcus salivarius* were more abundant in controls.
Olm (2019) [112]	28 to 32	-	Pre diagnosis	34 NEC 126 controls	Genome-resolved metagenomic analysis	Significantly more *Klebsiella pneumoniae* in NEC. Replication rates of all bacteria, especially *Enterobacteriaceae* family, significantly higher 2 days before NEC onset.
Lindberg (2020) [97]	23–27	485 to 1,026	Pre diagnosis	5 NEC 5 controls	16S rRNA gene sequencing	Pseudomonadota and bacteria belonging to the class Gammaproteobacteria were more abundant among NEC infants. Control infants showed greater abundance Bacillota.
Duan (2020) [113]	*≤* 37	-	Pre and post diagnosis	28 NEC 30 controls	PCR-DGGE	NEC stool samples had lower diversity. *Bacteroides* and *Klebsiella* was found in more NEC samples, whereas *E.coli*,* Bifidobacterium* and *Lactobacillus* were found in more control samples.
Fu (2021) [117]	*≤* 33	-	Pre and post diagnosis	15 NEC 15 controls	16S rRNA gene sequencing	Bacteroidota and Actinomycetota were more abundant at birth in infants that later developed NEC. Gammaproteobacteria, Enterobacteriaceae and Clostridiaceae are less abundant in stool of infants with diagnosed NEC.
Shaw (2021) [56]	< 32	-	Pre diagnosis	11 NEC 11 controls	qPCR	No difference in overall bacterial abundance between NEC and control samples.
					Shotgun metagenomic sequencing	Stool samples from NEC infants show either high proportions of LPS-expressing bacteria or low frequency of CpG motifs within their bacterial DNA.
Tarracchini (2021) [114]	23 to 39	900 to 3010	Post diagnosis	64 NEC 81 controls	Shotgun metagenomic sequencing	*E.coli* and *Enterococcus faecalis* were more abundant in stool samples from infants with NEC. *Streptococcus agalactiae* was more abundant in stool samples from control infants than those with NEC. *Staphylococcus epidermidis* and *Klebsiella pneumoniae* did not differ between NEC and control stool samples.
			Pre diagnosis	26 NEC 81 controls		Known pathogen species *K. pneumoniae*,* Proteus mirabilis*,* C. perfringens*,* C. neonatale*,* Pantoea dispersa and S.aureus* were more abundant in stool samples prior to NEC diagnosis.
Masi (2021) [98]	< 32	-	Pre diagnosis	14 NEC 34 controls	Shotgun metagenomic sequencing	NEC stool samples showed higher abundance of Pseudomonadota and lower abundance of Actinomycetota. *Enterobacter cloacae* were more abundant, while *Bifidobacterium longum* was less abundant in NEC stool samples.
Huang (2022) [99]	-	-	Pre and post diagnosis	9 NEC 10 controls	16S rRNA gene sequencing	Composition differs between NEC and controls. In NEC infants the Pseudomonadota phyla was most abundant and there was reduced abundance at the genus level of *Enteroccocus*,* Streptococcaceae* and *Lactobacillus.*
Du (2023) [100]	*≤* 34	-	Pre and post diagnosis	16 NEC 16 controls	16S rRNA gene sequencing	While no difference in overall diversity was reported, at the phylum level abundances of Pseudomonadota increased and Actinomycetota decreased in NEC. At the genus level *Bifidobacterium* and *Lactobacillus* abundance decreased in NEC.
Zenner (2023) [120]	< 32	922 mean	Pre and post diagnosis	11 NEC 21 controls	16S rRNA gene sequencing	Prior to NEC diagnosis no difference was observed between NEC and controls. At day 21 of life, increased abundance of *Escheria-Shigella* and lower *Enterococcus* abundance seen in NEC
Aires (2023) [94]	< 32	-	Post diagnosis	37 NEC 66 controls	16S rRNA gene sequencing	No difference in abundance or diversity between NEC and controls.
					Culture	Antibiotic susceptibility of *C. butyricium*,* C. neonatale*,* E. coli* strains isolated from NEC and controls did not differ.
Wang (2024) [121]	26 to 43	700 to 4300	Post diagnosis	18 surgical NEC 18 medical NEC 18 controls	16S rDNA gene sequencing	*Enterobacteriaceae* was enriched in intra-luminal stool collected from jejunum during NEC resection surgeries. *Staphylococcus* and *Stenotophomas* was enriched in intra-luminal stool collected from jejunum during control surgeries. *Granulicatella* was enriched in nappy stool collected from infants with NEC under medical management.
Thanert (2024) [88]	≤ 37	≤ 1500	Pre diagnosis	48 NEC 96 controls	Shotgun metagenomic sequencing and transcriptional analysis	No metagenomic or transcriptional difference between NEC and controls microbiome. Persistent low diversity and functional immaturity is seen in only late-onset NEC (*≥* 40 days of life) compared to controls.
Chae (2024) [105]	22 to 40	-	Pre diagnosis	46 NEC/feed intolerance 264 controls	qPCR	Significantly lower diversity in NEC/feeding intolerance infants despite no change in overall number of identified species. Lower abundance of Actinomycetota phyla, *Streptococcus* and *Bifidobacterium* genus in NEC/feeding intolerance infants.
El Manouni El Hassani (2025) [104]	*≤* 30	-	Pre diagnosis	56 NEC 56 controls	IS-pro™	*C. perfringens* was detected more often in NEC stool samples. There was no difference in abundance or diversity between stage II or IIIA NEC and control stool samples. Stage IIIB NEC stool samples were less diverse, with reduced Bacteroidota, Bacillota, Actinomycetota, Fusobacteria and Verrucomicrobiota.
Wang (2025) [103]	< 37	-	Pre diagnosis	13 NEC 13 controls	16S rRNA gene sequencing	Bacillota decreased and Gammaproteobacteria increased in the stool of preterm infants with NEC.

## Intestinal dysbiosis and inflammatory responses in NEC

**Fig. 1:**
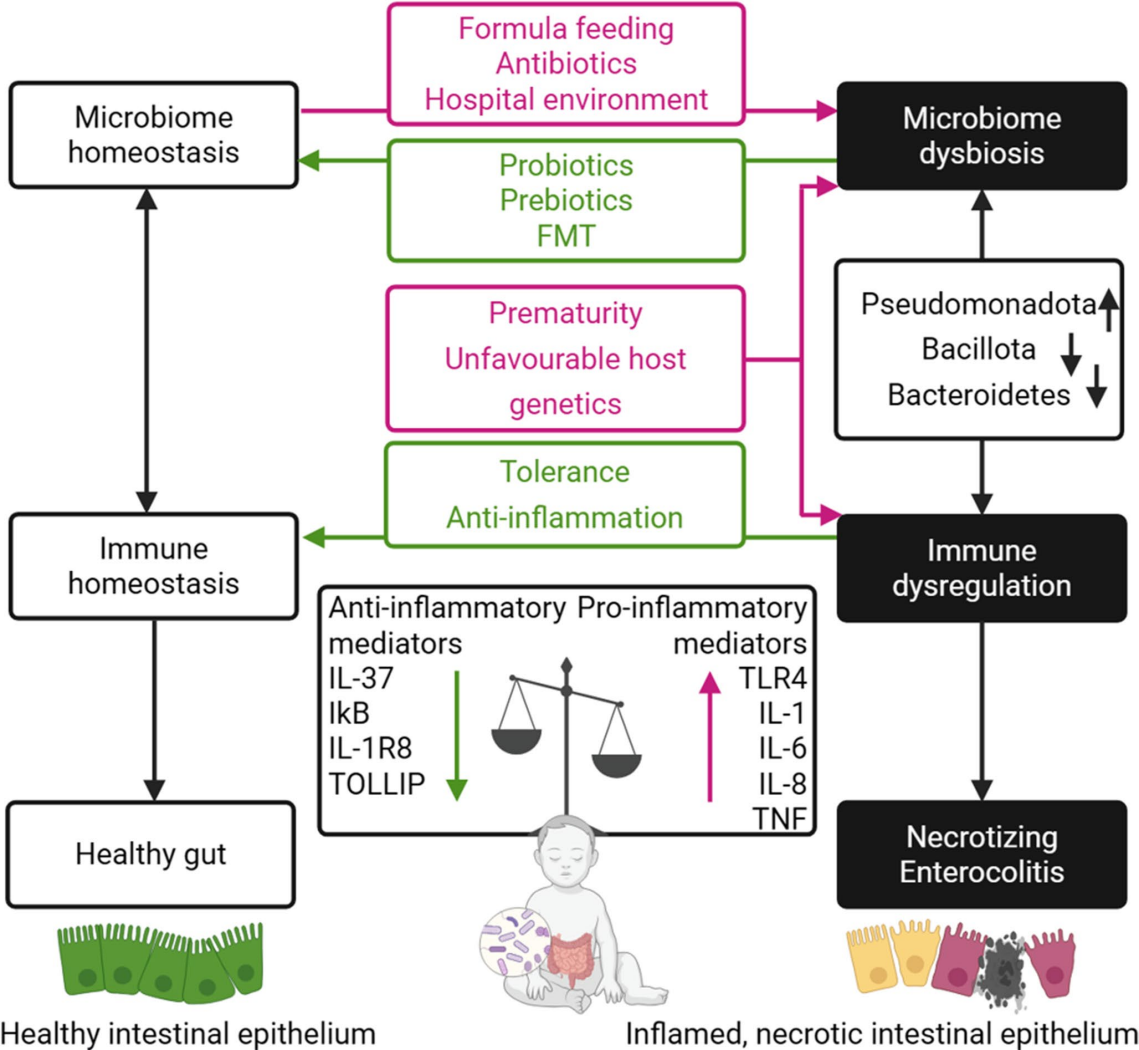
Microbiota-mediated immunopathogenesis of NEC. Risk factors in early life can lead to gut dysbiosis, increasing the abundance of TLR4, pro-inflammatory cytokines and inflammatory mediators, resulting in inflammation. Due to a prematurity-associated reduced abundance of IκB, IL-1R8 and TOLLIP, inflammation can quickly get out of control leading to the inflammation and tissue damage seen in NEC. This is not a comprehensive list of inflammatory mediators and work is ongoing to characterize the pathoimmunology of NEC. Red arrows/boxes indicate harmful effects and green arrows/boxes indicate beneficial effects in NEC. Created in BioRender. Abbreviations: necrotizing enterocolitis (NEC), fecal microbiota transplant (FMT).

Intestinal dysbiosis has been suggested to precipitate inflammation and subsequently drive inflammatory diseases including NEC. This theory rests on findings that the intestinal microbiota differs between NEC and control infants, and that antibiotic treatment [126, 127], histamine 2 blockers [128], antacid therapy [129] and formula feeding [20], each known to alter the intestinal microbiome, increased the risk of NEC. Significant associative evidence suggests that one of the main drivers of excessive inflammation-induced tissue damage preceding NEC is the interactions between an abnormal microbiome and the preterm immune system (Fig. 1) [77]. Furthermore, a balanced stable microbiome has been linked to health and reduced inflammation, suggesting that microbiome instability can contribute to inflammation. While opportunistic pathogens such as *Enterobacter cloacae* [98, 130], *E. coli* [114, 119], *Staphylococcus aureus* [98, 102] and *Klebsiella spp.* [106, 111–114] have been associated with NEC, there is a lack of experimental validation demonstrating causation or functional mechanisms of microbiota-driven inflammation in NEC or model systems. The exceptions here are *Cronobacter sakazakii *and *Clostridium* species, which are discussed in further detail below.

### Inflammation induced by *Cronobacter sakazakii*

In 1998, a NEC outbreak arose from Pseudomonadota species, *C. sakazakii*, via formula contamination [131]. Experimental validation of *C. sakazakii* infection in murine NEC revealed that increased dendritic cell (DC) activation and decreased macrophage/neutrophil recruitment in the intestine precipitated NEC [132, 133]. In vitro infection with *C. sakazakii* led to increased abundance of IL-1β, TLR4, the inflammasome components NLRP3 and caspase-1, and the adapter protein MyD88 in human colon HT-29 cells [134]. *C. sakazakii* also induced cell death in jejunal J774A.1 enterocytes and intestinal damage in murine NEC [134]. Neutrophil- and macrophage-mediated phagocytosis conferred protection against *C. sakazakii*, their depletion from the intestine lamina propria worsened murine NEC and was accompanied by increased abundance of DCs, pro-inflammatory cytokines and inducible nitric oxide synthase [133].

### Inflammation associated with *Clostridium* species

*Clostridium* species of the Bacillota phylum, such as *C. neonatale*,* C. butyricum* and *C. perfringens*, may contribute to NEC pathogenesis [108, 128, 135, 136], although the mechanistic pathways through which *Clostridium* species promote intestinal inflammation remain elusive. *C. perfringens* abundance was increased in NEC piglets [137] and activation of TLR4 by *Clostridia* may occur in NEC as infection induced host immune responses involving TLR4/MyD88/NF-κB signaling pathways in piglets [138]. Complementing in vitro experiments showed induction of the pro-inflammatory *CCL5* and *IL8* and the anti-inflammatory *IL1RN*, *NFKBIA* and *TNFAIP3* due to activation of TLR2 by *C. perfringens*’ cell wall component peptidoglycan when incubated with intestinal porcine IPEC-J2 enterocytes [137]. Heat-killed *C. butyricium* and *C. neonatale* isolated from NEC infants induced IL-8 in human Caco-2 colon cancer cells, but the same bacteria isolated from control infants did not [139].

### Insufficiency of anti-inflammatory responses

Actinomycetota, Bacillota and Bacteroidota are reportedly less abundant in NEC compared to non-NEC infants [30, 97, 112, 140–143]. Despite an overall reduction in Bacillota abundance, the enrichment of certain *Clostridium* species may promote NEC pathogenesis, indicating the complexity of the role of microbiota in NEC. Phylum level classification is too coarse to discern specific inflammatory properties. Bacteria from the Bacillota, Bacteroidota and Actinomycetota phyla can initiate inflammatory pathways through their microbial-associated molecular patterns, but also induce Tregs and other regulatory mechanisms, possibly dampening inflammation and augmenting tolerance [144, 145]. Low level production of short chain fatty acids (SCFAs) by bacteria, including *Bifidobacterium infantis*, also contributes to mucosal integrity [146].

## Modeling NEC

Studying NEC in human infants is not straightforward. Only minimal blood volumes and small intestinal resection specimens can be obtained. Stool usually collected from nappies, though more accessible, may not accurately represent the microbiome at the site of disease. Thus, modeling NEC is key to advancing knowledge.

Most current NEC pathogenesis evidence stems from animal studies in rodents, rabbits, pigs, and non-human primates (NHPs) [23, 147]. Although animal models provide important mechanistic insights, it is critical to understand their limitations in terms of human disease transferability, including differences in intestinal development, enteral nutrition, disease pathology [147] and particularly microbial composition [148, 149]. Caution is needed in interpretation of animal microbiome data, as the overlap with the human microbiome varies (e.g. only 3–4% overlap between humans and mice) [148, 149]. Furthermore, replicating the multifactorial origins of NEC in animal models is challenging.

### In vivo models

#### Rodents

In 1974, Barlow and colleagues used newborn rats as the first animal model of NEC. Control pups received rat breastmilk for 72 h, while NEC was induced via formula feeding combined with daily 3–5 min asphyxia, with or without *Klebsiella pneumoniae* gavage-inoculation [150]. Asphyxia mimicked hypoxic episodes, common in preterm infants. The rat model reproduced human NEC symptoms, including abdominal distention, lethargy, bloody stools, and necrotic intestines [150] but lacked hallmark features of human NEC, pneumatosis intestinalis or portal venous gas. To mimic vasoconstriction and ischemic-reperfusion injury known to NEC, modifications such as 5 min of 100% O_2_ reoxygenation followed by asphyxia [151] and 5 min of cold stress at 1°C were included [152].

Subsequent NEC rat models applied hypoxia (usually 5% O_2_/95% N_2_ for 3–10 min) [153–156] in combination with formula feeding to induce NEC-like lesions in the intestines of preterm, term and 2-3-day old rats [153–156]. Since NEC was often mild in this model, LPS administration was added to mimic intrauterine inflammation via TLR4 activation [157]. This three-hit-model enabled testing of microbiome-modulation, e.g. human milk oligosaccharides (HMOs) [158], fecal microbiota transfer (FMT) [159] or administration of probiotic *Lactobacillus reuteri*, *Bifidobacterium longum infantis* [160], *Bifidobacterium adolescentis* [161] and *Bifidobacterium bifidum* [147], with varying clinical outcomes.

Despite the challenge of smaller pups, mouse NEC models have gained popularity over rat models due to the advantage of using genetically modified strains. Notably, term murine pup intestines are immature at birth, and only reach full maturity by postnatal day 28 [162]. NEC-like pathology has been introduced in newborn [22, 148], 3-day [163], 7-10-day [164, 165] or in 20-36-day old [166] mice via exposure to hypoxia/asphyxia, cold stress and formula feeding. NEC pathogenesis increased with feeding formula spiked with LPS or stool-derived bacterial slurry from NEC-infants, reinforcing the role of microbial dysbiosis.

In summary, rodent models of NEC have improved our understanding of the microbiome’s role in NEC pathogenesis and potential treatments. However, inconsistencies between models and inter-species microbial differences limit the application of those findings.

#### Other small animals

While rodents represent the most employed small animal NEC model, rabbits, hamsters, chickens and gnotobiotic quails are also available [167]. Rabbits require the small intestine or colon to be surgically segmented and injected with casein and calcium gluconate to mimic human NEC [168–170]. This time- and labour-intensive model was used to link NEC with low pH [169], low lactase, organic acids, and high levels of histamine and O_2_-derived free radicals [171, 172]. Other experiments involving preterm C/S-delivered rabbits have shown that formula supplemented with *Enterobacter cloacae* combined with poor intestinal motility induced NEC-like pathology [173].

Less common are germ-free quails and chickens to study specific bacterial species isolated from human NEC stool [174, 175]. Oral inoculation of *Clostridium beijerinckii*, in 2-50-day old axenic chickens [174] caused cecal inflammation and necrosis. Gnotobiotic quails orally inoculated with *Clostridium butyricium*, which produces butyric acid, developed intestinal NEC-like lesions [175].

#### Large animals

Piglets delivered at ~ 90% full gestation (104 days) represent an attractive model for NEC due to gastrointestinal maturity similar to preterm infants born at 30–32 weeks GA [176]. In both term and preterm piglets, intestinal intraluminal casein injection induced NEC-like lesions [177, 178]. In term piglets, mesenteric artery and lymphatic occlusion caused necrosis, immune infiltration, mucosal thinning and epithelial barrier disruption, mimicking human NEC [179], as did a combination of hypoxia and cold stress [180]. In weanling pigs, intraperitoneal LPS injection or formula supplementation induced intestinal epithelial necrosis reminiscent of NEC [181]. These studies implicated diet and microbial fermentation of undigested carbohydrates (producing CO_2_ and H_2_), as causes of pneumatosis intestinalis and portal venous gas in NEC [182].

Non-human primates (NHPs) most closely resemble humans in modelling NEC. However, NHP models are extremely expensive and rarely employed in NEC research [147]. Baboons, delivered preterm by C/S, maintained in intensive care using human protocols and fed infant formula for 21 days, developed NEC at an incidence of 5% [64], with a similar clinical presentation as babies affected by NEC (abdominal distention, intestinal inflammation, haemorrhage). As in human preterm infants, intestinal TGF-β_2_ was decreased in preterm vs. term baboons and further decreased in NEC [64]. Additionally, NHPs and piglets share microbiome similarities to humans at a phylum level following birth [183, 184].

In summary, NHPs followed by piglets come closest to human infants in terms of intestinal maturity, physiology, nutritional requirements and likely also their microbiome and immune system development and function. However, large animal models carry high costs and require specialized facilities and training, rendering their use unfeasible for many scientists.

### In vitro and ex vivo models

#### Cell culture

Barrier function, inflammatory signaling and cell-cell or bacteria-cell interactions have been investigated in Caco-2 and HT-29 adult human colon carcinoma epithelial cells, H4 human fetal small intestinal cells, IEC-6 rat small intestinal epithelial cells and IPEC-J2 jejunal porcine enterocytes [137, 185]. Caco-2 monolayers were used to study translocation of two early intestinal colonizers, *E. coli*, which was increased in immature, undifferentiated cells and *C. difficile* mediated translocation through toxin B opening of tight junctions [186, 187]. Although valuable to investigate specific cellular responses and barrier functions, single cell-type in vitro cultures cannot mimic crosstalk between immune, epithelial, and endothelial cells in vivo and immortalized cell lines often have altered immune responses and barrier functions when compared to primary cells.

#### Primary tissue xenografts

Primary human fetal small intestinal tissue without mesentery can be subcutaneously implanted into young immunocompromised mice and harvested at different maturity stages for organ culture [53, 188, 189]. Xenograft models have significant benefits over single cell culture: they are generated from primary human tissue, have relevant immune responses and maintain structural integrity important for microbiota interaction [53]. Following LPS- or IL-1β-stimulation, exposure to *Bifidobacterium infantis-* and *Lactobacillus acidophilus*-conditioned media attenuated excessive IL-8 and IL-6 production [188], likely via reduced TLR4/MyD88-mediated NF-κB activation, indicating potential beneficial effects of probiotics [188].

#### Organoids, organoid-microbiota co-cultures, and organ-on-a-chip

Organoids are 3D-structures grown from stem cells that self-organize through cell sorting and spatially restricted lineage commitment [190]. Unlike primary tissue explants, they survive in long-term culture (weeks) and intestinal organoids spontaneously form an intact epithelial layer and a crypt-villus structure. Organoids from pluripotent stem cells, fetal intestines and infant tissue resections resemble immature intestines and can be used to model NEC [191].

Intestinal organoids from mouse pups exposed to hypoxia plus LPS or LPS alone for 48 h [192, 193] exhibited increased *Il6*,* Tnf* and *Tlr4* expression and disrupted tight junctions [192, 193]. Breastmilk components, including HMOs, exhibited protective effects in these organoid NEC models [191, 193]. In human neonatal intestinal organoids, LPS increased *TNF*,* IL1B*,* TLR4* expression and apoptosis [194, 195], while microbial SCFAs reduced inflammation in IL-1β-stimulated fetal small intestinal organoids [196].

Further, organoids can be co-cultured with bacterial species from NEC infant stool, e.g. uropathogenic *E. coli* [197] and *Akkermansia mucinophilia* [198] species, by micro-injection into the organoid lumen, enabling interaction with epithelial cells [199]. This enables functional studies of specific bacteria or stool-isolated bacterial communities and their role in NEC pathology.

The novel microfluidics-based gut-on-a-chip (GoC) technology models the in vivo-intestinal micro-environment by creating separate luminal and submucosal compartments. GoC facilitates co-cultures with bacteria and immune cells via microfluid channels [200, 201] without needing to micro-inject bacteria. GoC models are promising to study inflammation and host microbiome interactions with commensal bacteria in NEC.

### NEC model summary

Various NEC models, ranging from low-throughput large animal models to small animal models, high-throughput cell line studies and advanced in vitro models such as GoC offer unique advantages for studying different disease aspects. However, when investigating the microbiome in NEC, substantial cross-species differences must be considered. Microbiome data from non-human models that do not use gnotobiotic approaches or fail to look beyond microbial diversity must be interpreted cautiously.

## Modulating inflammatory responses in NEC using microbiota-targeted intervention

Several beneficial microbes have been investigated to prevent or ameliorate NEC outcomes. Strategies, such as administering prebiotics, probiotics, FMT or live biotherapeutics, aim to achieve an anti-inflammatory and tolerogenic intestinal environment.

### Prebiotics

Prebiotics are defined as “a substrate that is selectively utilized by host microorganisms conferring a health benefit” [202]. Notably, human milk has prebiotic properties; it is rich in bioactive molecules, such as carbohydrates, proteins, fats, cytokines and Ig, that promote healthy microbial colonization and favourably regulate immune and inflammatory responses [203]. The risk of NEC can also be reduced by feeding breastmilk to neonates [204]. Beneficial effects include inhibiting signaling via TLR2 (soluble TLR2, soluble CD14), TLR4 (soluble CD14, lactadherin, lactoferrin, 2’-fucosyllactose), TLR3 (3’-galactosyllactose) and TLR7 (β-defensin 2) [203]. Meta-analyses have found that while some prebiotics (oligosaccharides, inulin, lactulose, lactoferrin) reduced mortality and rates of sepsis, there was no significant difference in NEC morbidity [40, 205]. Other prebiotic components of human breastmilk (e.g. maternal Ig, HMOs, and arginine) have also been suggested to reduce NEC severity and to alter microbial composition [206–209]. While many other components of breast milk likely have prebiotic effects, here we will focus on the microbiome and immune modulation induced by HMOs and maternal Ig.

#### Human milk oligosaccharides (HMOs)

After lactose and lipids, HMOs are the third-most abundant ingredient in human breastmilk, 13% of which are sialylated-HMOs (sHMOs) [210]. In colostrum, HMOs are more concentrated (20–25 g/L) than in mature milk (5–15 g/L) [211], however, infant formula only contains small amounts (100 mg/L) [212]. Many HMOs are indigestible for infants [213], but as the predominantly protective *Bifidobacteria* and *Bacteroides* have enzymes to metabolize HMOs, they selectively support their own intestinal colonization [214]. Thus, the low HMO quantities in formula were proposed to contribute to microbial dysbiosis and inflammation in formula-fed infants.

Approximately 200 structurally different HMOs are known, however, the functional role of different HMOs are largely unknown. Their composition in breastmilk is individual to each mother and varies throughout the lactation period. The best studied HMO is 2′-fucosyllactose, as it is found in 80% of all women’s breastmilk at a concentration of ~ 2.5 g/L. Microbial communities of young children incubated with pooled HMOs showed significant compositional shifts, including increased abundance of potentially beneficial *Bacteroides*, providing evidence that HMOs can directly alter the microbiome [215]. Animal models of NEC concluded that sHMOs may help to reduce the risk of NEC in premature infants [216]. Administering HMO-containing formula decreased proinflammatory markers, including CXCL15 (murine IL-8 homolog), in the serum and ileum of NEC-affected mice [206], reduced TLR4 protein abundance, NF-κB signaling, and restored proliferation of intestinal crypt cells in the ileum and in organoids [206]. Further, the NEC incidence in rats was reduced by sHMOs alongside TLR4, NLRP3 and caspase-1 levels [158].

Importantly, sHMO concentrations are lower in NEC infants’ compared to healthy infants’ maternal milk [98], and increased numbers of NLRP3- and caspase-1-positive lamina propria cells were observed in intestinal biopsies of NEC patients [158]. These findings suggest that sHMOs support the development of a beneficial microbiome, reduce inflammation and prevent NEC.

#### Maternal immunoglobulin (Ig)

IgA antibodies, secreted into the intestinal lumen and into breastmilk (secretory (s)IgA) [217], influence the composition of the intestinal commensal microbiota and bind to and protect against pathogens by preventing their recognition by TLRs [144, 217]. The intestinal sIgA abundance is lower in preterm infants than in their term peers. However, there is ample sIgA in maternal milk [144, 218], and it was surmised that the lower abundance of sIgA in formula-fed preterm infants predisposes them to NEC [217, 219]. Indeed, the lack of sIgA in formula promotes the activation of inflammatory pathways in response to bacterial colonization in preterm infants, thus likely driving NEC [53, 59, 188]. Notably, human NEC was associated with a reduction in IgA-bound bacteria; and most non-IgA-bound bacteria belonged to the potentially harmful *Enterobacteriaceae* family [207]. The lack of IgA coverage of pathogenic bacteria allows increased interaction with the reactive preterm immune system and bacterial translocation, potentially driving more severe inflammation. As seen in a murine NEC model, wild-type pups fed by IgA-deficient dams were more susceptible to NEC than those fed by IgA-producing dams, with NEC pathology similar to that of formula-fed wild-type pups [207]. Notably, Gammaproteobacteria-specific IgA partially facilitated the transition from premature to mature intestinal microbiota in mice [220].

In addition to IgA, maternal IgG provides passive immunity against enteric pathogens while humoral immunity develops, but is transferred mainly during the last trimester of pregnancy [221]. Hence, preterm infants are deprived of the protective capacities of IgG [221]. However, in a clinical trial, oral IgG failed to reduce NEC incidence [38]. Likewise, in another clinical trial, formula supplemented with IgA/IgG (73%/26%) failed to reduce NEC incidence and severity in human neonates [222], and oral gentamicin was found to be more effective at preventing NEC compared to oral IgA/IgG administration alone [223]. One major limitation of these trials was, however, that the effect of sIgA [217], which, due to the secretory component is more stable in the luminal environment, was not tested. Likewise, no clinical trial to date has evaluated the effects of IgA alone.

### Probiotics

Probiotics are defined as “live microorganisms which when administered in adequate amounts confer a health benefit on the host” [224], however, most probiotics do not colonize the intestine themselves but rather support the growth of other beneficial bacteria by improving barrier function and anti-inflammatory responses [225]. Studies in very (< 32 weeks GA) and extremely (< 28 weeks GA) preterm infants have shown that probiotic administration alters microbial diversity and composition [226, 227]. Probiotics vary in composition, often containing only a few microbial strains, and are rather simplistic compared to the vast diversity of species that colonize the infant’s intestine [225]. Lack of standardization and strain-level characterization also often creates batch-to-batch variation, complicating interstudy comparisons, including the interpretation of meta-analyses [228]. Despite some randomized controlled studies reporting minimal benefit of probiotics [229–232], most prospective cohort studies [233, 234], retrospective studies [235–237], randomized controlled trials [238–240] and meta-analyses [229, 241–244], predominantly investigating the effects of *Lactobacilli*, *Bifidobacteria* and *Bacteroides*, have suggested that probiotics do reduce the incidence of NEC. Further details regarding the anti-inflammatory effect of probiotics on the immune system in NEC models can be found in Table 2. Overall, current understanding is impeded by substantial heterogeneity regarding strains, dose, mode and timing of administration and outcomes evaluated. Considering these circumstances, it is unsurprising that evidence is somewhat conflicting.

In 2021, the American Academy of Pediatrics published a clinical report recommending against routine administration of probiotics in preterm infants, especially when birth weight is < 1000 g, citing concerns about the lack of a Food and Drug Association (FDA)-approved probiotic alongside conflicting safety and efficacy data [245]. Following a report of fatal *Bifidobacterium longum* probiotic sepsis, the FDA also released a statement warning of invasive disease risk in preterm infants receiving probiotics [246]. In response, the European Society of Paediatric Gastroenterology, Hepatology and Nutrition, alongside the European Foundation for the Care of Newborn Infants, acknowledged the need for future high-quality research, while advocating for the continued use of probiotics in preterm infants as documented benefits outweigh reported rare adverse effects [247]. A comprehensive 2025 meta-analysis of probiotic use including 20,323 preterm infants found probiotic sepsis occurred at a rate of less than 0.04% and NEC incidence decreased (relative risk 0.60, 95% confidence interval 0.51–0.71) [248]. Despite documented benefits, research into how probiotic species interact with the immune system to prevent inflammation is ongoing. Thus, the concept of probiotic treatment likely has not yet achieved its full potential.

**Table 2:** Pre-clinical evidence of probiotics modulating inflammatory responses from 2013 to 2024. Different probiotic strains were administered to mice, pig, rats, or cultured cells with or without inflammatory stimulation with produced changes in mRNA expression, protein abundance and/or cell proportions. Abbreviations: lipopolysaccharide (LPS), interleukin (IL), messenger ribonucleic acid (mRNA), single Ig IL-1-related receptor (SIGIRR), toll interacting protein (TOLLIP), regulatory T-cells (Tregs), toll-like receptors (TLR), nuclear factor kappa-light-chain-enhancer of activated B-cells (NFκB), chemokine (C-X-C motif) ligand 1 (CXCL), nitric oxide synthase (NOS), tumor necrosis factor (TNF), interferon (IFN), interleukin-1 receptor-associated kinases (IRAK), primary intestinal epithelial cells (P-IEC)

Probiotic strains	Host species	Sample type	Inflammatory stimuli	Modulation of immune mediators in response to probiotics		Mechanism of action of probiotics	References
				Increased	Decreased		
*Bifidobacterium infantis* and *Lactobacillus acidophilus*-conditioned media	Human	Immature human intestinal xenografts	LPS + IL-1α	mRNA: *SIGIRR* and *TOLLIP*	mRNA: *TLR2*, *TLR4* Protein: IL-8, IL-6	-	Ganguli (2013)[188]
	Human	Primary enterocyte culture of NEC tissue	LPS + IL-1α	mRNA: *SIGIRR* and *TOLLIP*	mRNA: *TLR2*, *IL8*, *IL6*	TLR2-mediated anti-inflammatory activity	
DNA from *Lactobacillus rhamnosus* HN001	Mouse	Ileum	Formula + hypoxia	-	mRNA: *Nos2*	TLR9-mediated reduction of NEC severity and inflammation	Good (2014)[165]
	Piglet	Ileum	Formula	-	mRNA: *NOS2*	-	
	Human	Ileum	LPS	-	mRNA: *NOS2*, *IL6*; NF-κB translocation	TLR9-mediated inhibition of TLR4 signaling	
*Bifidobacterium longum* subsp. *infantis*	Rat	Ileum	Formula + hypoxia + cold stress	-	mRNA: *Il6*, *Cxcl1*, *Tnf*, *Il23* Protein: iNOS	-	Underwood (2014)[160]
*Lactobacillus reuteri* DSM 17938	Mouse	Ileum	Formula + hypoxia + cold stress	Tregs	Effector/memory T cells	-	Liu (2014)[249]
*Bifidobacterium* microcapsules	Rat	Ileum	LPS + formula		Protein: TLR2, TLR4, NF-κB p65	-	Zhou (2015)[250]
*Bifidobacterium longhum* subsp *infantis* conditioned media	Human and mouse	H4 cell line; human fetal small intestinal xenografts; mouse fetal ileum; primary NEC enterocytes	IL-1β	-	mRNA: *IRAK2* Protein: IL-6; phospho-c-Jun and phospho-c-Fos (in H4 cells)	TLR4-mediated anti-inflammatory activity	Meng (2016)[251]
*Bifidobacterium adolescentis*	Rat	Intestine	Formula + asphyxia + cold stress	mRNA: *Tollip*,* Sigirr*	mRNA: *Tlr4*	-	Wu (2017)[161]
*Bacteroides fragilis* and/or PSA	Mouse	Intestine	IL-1β	-	Protein: IL-8	-	Jiang (2017)[252]
PSA of *B. fragilis*	Human	H4 cell line; fetal enterocytes from resected NEC small intestine	IL-1β	-	Protein: IL-8	TLR2- and TLR4-dependent inhibition of AP-1 genes *JUN* and *FOS*	Jiang (2017)[252]
*Lactobacillus reuteri* DSM 17938	Mouse	Ileum; spleen (DCs)	Formula- + hypoxia + cold stress	FoxP3^+^ Tregs; Tolerogenic DCs	% of activated effector CD4^+^ T cells Protein: IL-8 IFN-γ, IL-1β	TLR2-mediated DC recognition and DC-priming of Tregs	Hoang (2018)[253]
PSA of *Bacteroides fragilis*	Human	H4 (fetal small intestinal cell line)	IL-1β	-	mRNA: *IL8* Protein: IL-8	Zona pellucida protein 4 (TLR2- and TLR4-dependent)-mediated decrease in expression of IL8	Gorreja (2019)[254]
*Lactobacillus reuteri* DSM 17,938	Mouse	Small intestine	-	-	FOXP3^+^ Tregs	-	Liu (2019)[255]
*Bacteroides fragilis* strain ZY-312	Rat	Serum; faeces	*Cronobacter sakazakii*	Protein: EGF (serum);	Protein: TNF, IFN-γ (serum);	-	Fan (2019)[256]
ILA *from Bifidobacterium longhum* subsp *infantis*	Human and mouse	H4 cell line; intestine (mouse)	IL-1β	-	Protein: IL-8, MIP2	Aryl hydrocarbon receptor mediated anti-inflammatory response	Meng (2020)[257]
*Lactobacillus rhamnosus GG*	Mouse	Intestine	Formula	mRNA: *Sigirr*,* A20*	mRNA: *Icam1*,* Il1b*,* Il8*,* Tlr4*,* Irak1*	Induced expression of TLR inhibitors inhibits TLR-dependent inflammation	Cuna (2021)[258]
*Bifidobacterium infants*,* Lactobacillus acidophilus*,* Enterococcus*, and *Bacillus cereus*	Mouse	Ileum	Formula + hypoxia + cold stress	-	mRNA: *Tnf*,* Il1b*,* Il6*	PXR suppression of JNK signaling enhances barrier function	Zhao (2021)[259]
ILA *from Bifidobacterium longhum* subsp *infantis*	Human and mouse	H4 cell line; intestine (mouse)	IL-1β	mRNA: *Il6*,* Stat1/STAT1*	mRNA: *Il8*,* Paf1*,* Pafr*,* Traf2*	Upregulation of *Stat1/STAT1* inhibits IL-1β driven inflammation	Huang (2021)[260]
*Ligilactobacillus salivarius YL20*	Mouse	Small intestine	*Cronobacter sakazakii*	-	mRNA: *Il1b*,* Il6*,* Tnf* Protein: IL-1β, IL-6, TNF	-	Wang (2022)[261]
*Lactobacillus rhamnosus* or L*actobacillus acidophilus* and *Bifidobacterium bifidum*	Human	Cord blood mononuclear monocytes	LPS	% of TLR4 + monocytes, Protein: IL-1β, TNF	-	Stimulation of immune cells may improve their ability to fight infection	Rückle (2022)[262]
*Lactobacillus rhamnosus* GG	Mouse	Ileum	Prenatal antibiotics + formula	mRNA: *Gpr81*,* Axin2*,	-	Activation of Wnt signaling increases intestinal proliferation	Cuna (2023)[263]
*Bifidobacterium breve AHC3*	Rat	Ileum	Formula + LPS + hypoxia + cold stress	Protein: IL-10	Protein: TNF	Inhibition of iNOS expression protects against intestinal injury	Lin (2023)[264]
*Lactobacillus reuteri DSM 17938*	Mouse	Ileum	Formula + hypoxia + cold stress	-	mRNA: *Tnf*,* Il1b* Protein: TNF, IL-1β	Improves gut barrier function, by reduced pro-inflammatory cytokine production	Lai (2024)[265]
*Bacteroides fragilis*	Rat	Colon	LPS + hypoxia + cold stress	mRNA: *Il6*	Protein: FXR, NLRP, IL-1β	*Bacteroides fragilis* metabolizes bile acids and inhibits FXR-NLRP pathway	Chen (2024)[266]
*Limosilactobacillus fermentum CECT5716*	Human	P-IECs	LPS	-	Protein: IL-6	TLR9 mediated anti-inflammatory activity	Hedegger (2024)[267]

#### *Lactobacillus *spp

*L. reuteri* increases tolerogenic DC and FoxP3^+^ Treg numbers, while reducing effector CD4^+^ T cells and IFN-γ and IL-1β abundance by inhibiting TLR2 [253], thus reducing murine NEC severity. Likewise, increased intestinal Foxp3^+^ Tregs following administration of *L. reuteri* strain DSM-17938 was suggested to confer protection against NEC by decreasing the mRNA and protein abundance of *Il6*,* Tnf*,* Tlr4* and *Nfkb*, while increasing anti-inflammatory *Il10* in the intestine of NEC-affected rats (Table 2) [255]. Feeding *L. reuteri* to healthy dam-fed mice increased bacterial diversity and relative abundance of the Clostridiales order and decreased relative abundance of the genera *Bacteroides*,* Sutterella*,* Akkermansia* and *Ruminococcus* compared to controls [255]. However, how changes in relative bacterial abundance facilitate or prevent NEC remains unclear, pointing to the limited microbiome overlap between human and mice [149], especially as many *Lactobacilli* species only colonize the human intestine in the absence of competition or following microbiome perturbation. Nevertheless, a randomized, double-blinded, placebo-controlled trial showed that *L. reuteri* supplementation caused increased bacterial diversity and reduced abundance of *Enterobacteriaceae* and *Staphylococcaceae* in extremely low birth weight infants, however, no significant positive effect on NEC incidence was reported [268].

Similarly, the *Lactobacillus rhamnosus* strain HN001 reduced NEC severity in mice and piglets [165]. In intestinal tissue from humans and in cultured IEC-6 rat enterocytes, *Lactobacillus rhamnosus* HN001 inhibited TLR4 signaling via TLR9 activation (Table 2) [165]. *Lactobacillus rhamnosus* strain GG reduced the intestinal injury severity in the colon of NEC-affected mice by augmenting IL-10 receptor-mediated signaling, causing STAT3-mediated induction of SOSC3, which prevented pro-inflammatory TNF and CCXL2 protein production [269].

#### *Bifidobacterium* spp

*B. adolescentis* reduced NEC severity in rats by decreasing *Tlr4* (Table 2) [161]. Similarly, *Bifidobacterium infantis*-conditioned medium or *Bifidobacterium* microcapsules decreased intestinal *TLR2* and *TLR4* mRNA in immature human intestinal xenografts [188], or their protein abundance in rats (Table 2) [250]. Interestingly, TLR4 was required for the inhibition of IL-6 by *B. infantis*-conditioned medium in enterocytes isolated from human NEC tissue and fetal murine intestinal tissue [251]. Being gram-positive, *Bifidobacteria* lack LPS but are thought to modulate TLR4 via yet unidentified secretory molecules [251]. In term newborn NEC rats, microcapsules of *Bifidobacterium* also reduced the abundance of NF-κB p65, while *B. longum infantis* reduced intestinal expression of the pro-inflammatory *Il6*,* Cxcl1*,* Tnf*,* Il23* and *Inos* (Table 2) [160]. In immature human intestinal xenografts and primary enterocyte cultures of NEC tissue, *B. infantis-* and *L. acidophilus*-conditioned media also increased anti-inflammatory mediators *IL1R8* and *TOLLIP*, and reduced IL-6 and IL-8 protein concentrations in a TLR2- and TOLLIP-dependent fashion (Table 2) [188]. Similarly, *B. adolescentis* reduced NEC severity by increasing *Il1r8* and *Tollip* mRNA in a rat NEC model [161]. A lack of anti-inflammatory mediators including IL-1R8 and TOLLIP leading to excessive inflammatory responses of NEC-affected epithelia is further supported by our own study in which intestinal IL-1R8 was decreased in NEC infants compared to healthy controls and infants that had recovered from NEC [22].

*B. infantis* found in breastmilk can also secrete the anti-inflammatory tryptophan metabolite ILA (indole-3 lactic acid) [257, 260] that reduced IFN-γ in immature human enterocytes and the colon and ileum of mouse pups [260]. Thus, ILA might also mediate the anti-inflammatory effects seen in response to *B. infantis*-conditioned media.

#### *Bacteroides* spp

The developmentally regulated gene *ZP4* (zona pellucida-protein 4) was implicated in the anti-inflammatory functions of *Bacteroides fragilis*’ surface component PSA (polysaccharide A), reducing IL-8 concentrations in H4 fetal intestinal cells in a TLR2- and TLR4-dependent manner (Table 2) [254]. Moreover, pretreatment with the *B. fragilis* strain ZY-312PSA ameliorated NEC severity and intestinal injury caused by *C. sakazakii* infection by inhibiting pro-inflammatory TNF and IFN-γ and the inflammasome (NLRP3, caspases 1/3), while increasing the anti-inflammatory IL-10 [256]. In rats, *B. fragilis* ZY-312 pretreatment, furthermore, prevented *C. sakazakii*-induced harmful microbiome changes including reduction of Bacteroidota and increase of Pseudomonadota species associated with human NEC (Table 2) [256].

### Fecal microbiota transfer (FMT) and fecal filtrate transfer (FFT)

Transfer of intestinal microbiota-containing fecal material from a healthy donor to a sick individual (rectally, orally or via a stoma) as whole fecal material (FMT) or filtered as FFT, leaving only bacteriophages, extracellular vesicles and bacterial products, has shown promise in numerous gastrointestinal conditions [270]. FMT is an established treatment for *C. difficile* infections [271], and has been suggested for the treatment of Ulcerative colitis [272], Crohn’s disease [271], insulin resistance [273] and NEC [159, 274–277]. In a rat NEC model, FMT from a healthy adult to a neonatal rat decreased IL-1β, TNF and IL-6 and intestinal neutrophil infiltration [159]. In piglets healthy colon, luminal fecal transplant increased the relative abundance of obligate anaerobes, mucosal resistance to bacterial adhesion, preserved goblet cell mucin stores and reduced the incidence of induced NEC [277]. Similarly, in murine NEC, FMT prevented tissue injury, decreased intestinal inflammation, improved barrier function via increasing claudin-7 (a tight junction protein), and improved NEC symptoms through reduced free oxygen radicals and increased nitric oxide [276]. Further, FMT reduced TLR4-mediated pro-inflammatory signaling and inflammatory cytokine abundance, and suppressed intestinal apoptosis [274], which decreased bacterial translocation across the intestinal barrier and increased the total intestinal bacterial number [274]. However, rectal FMT administration in preterm piglets following neomycin and amoxicillin-clavulanate antibiotic cessation conferred no protection against NEC, despite FMT administration or antibiotic treatment alone reducing NEC incidence [275]. Antibiotics may induce changes in the gut (altered pH, nutrient availability, immunoreactivity) or overgrowth of antibiotic-resistant species which hinder the beneficial effect of FMT-delivered species.

Despite FMT showing promise in animal NEC models, its translation to humans faces challenges. Given the differences between the adult and the neonatal intestinal microbiome, adult-sourced FMT is potentially unsafe for infants [278]. Sourcing healthy infant donors is extremely challenging, among low gestational age preterm infants athigh risk of NEC. Artificial FMT comprised of laboratory-produced microbial communities with known beneficial species or the manipulation of fecal material such as FFT, that has shown efficacy equal to FMT in piglets [279], may represent more clinically acceptable approaches in NEC. However, significant microbiome variation between individuals further complicates the identification of an ‘ideal’ microbiome for FMT or FFT and the efficacy of combination therapies needs careful evaluation.

## Conclusion

NEC is a devastating multifactorial disease that clouds the prognosis of preterm infants. Despite years of research, no gene or group of genes could be singled out as causative, and the variability of the pathogenetic processes leading to NEC and of its clinical course suggests that NEC may not be a single disease but rather a syndrome. The timing of bacterial colonization of the gastrointestinal tract also appears to be a critical factor in NEC development, with certain microbes interacting with the immune system differently to promote either inflammation or tolerance at different times of immune maturation.

As a result, gathering knowledge on precipitating factors and their interactions in driving NEC pathology is highly challenging, which in turn renders devising appropriate diagnostic tools (including biomarkers), developing preventive strategies and safe and effective treatments difficult. However, recent research has made substantial progress in understanding the intestinal dysbiosis and its interplay with the developing immune system in NEC, highlighting the translational promise of immune- and microbiota-based therapeutics. Unravelling the mechanisms of how intestinal bacterial colonization influences the developing immune system of preterm infants, and vice versa, could be the missing link that researchers have been seeking for years in their efforts to combat NEC and ameliorate the immense suffering wrought by this insidious disease.

## Data Availability

Data sharing not applicable to this article as all data generated or analysed are included in this published article.
